# Inhibition of PAK1 alleviates cerulein-induced acute pancreatitis via p38 and NF-κB pathways

**DOI:** 10.1042/BSR20182221

**Published:** 2019-03-01

**Authors:** Minghui Zhu, Yan Xu, Wenbin Zhang, Tianyi Gu, Daming Wang

**Affiliations:** Department of Emergency, Changzhou City No.1 People’s Hospital, Changzhou, Jiangsu Province, 213000, China

**Keywords:** Acute pancreatitis, FRAX597, Inflammation, NF-kB, PAK1, p38

## Abstract

Acute pancreatitis is a life-threatening disease accompanied by systemic inflammatory response. NF-κB and p38 signal pathways are activated in AP induced by cerulein. And PAKs are multifunctional effectors of Rho GTPases with kinase activity. In the present study, the function of P21-activated kinase 1 (PAK1) in AP was investigated, and found that PAK1 was up-regulated in pancreas of AP mice model, and led to NF-κB and p38 pathway activation. PAK1 inhibition by shRNA or small molecule inhibitor FRAX597 decreased NF-κB and p38 activity, also alleviated the pathological damage in the pancreas of AP mice model, including decreasing the amylase and lipase levels in serum, decreasing the levels of tumor necrosis factor-α, interleukin-6, and interleukin-1β in AP. These results suggested that PAK1 inhibition protects against AP by inhibiting NF-κB and p38 pathways, and indicated that PAK1 is a potential therapy to alleviate AP patients in clinic, and these need to be explored further.

## Introduction

Acute pancreatitis (AP) is a relatively common inflammatory disorder of the pancreas, leads to local and systemic complications [1]. And severe AP is potentially fatal and responsible for significant human morbidity and mortality worldwide. AP always begins with a local inflammation of pancreas, and then causes multiple organ dysfunction, such as lung, liver, and gut [2–4]. The links between pancreas injury and other organ dysfunction is systemic inflammation [4]. Many researchers have attempted to identify the initiation and aggravation of AP, but the mechanism is still poorly understood, and urgent to breakthrough to support effective treatment.

The p38 MAP kinases are a family of serine/threonine protein kinases, belong to MAPK superfamily, and have four isoforms. The p38 MAP kinases play important roles in cellular responses to external stress signals. Amongst four isoforms, p38α is the best studied and the most relevant kinase involved in inflammatory response to regulate the biosynthesis of key proinflammatory mediators, such as TNF-a and IL-1b [5]. Some studies showed that p38 is involved in AP [6,7] and regulated by P21-activated kinase 1 (PAK1) [8,9].

NF-κB is a transcription factor and is involved in cytokine production [10], cancer development [11–14], and response to inflammatory stimuli [15–17]. NF-κB consists of two proteins p50 and p65, and activated by p65 [18]. NF-κB activation leads to the expression of pro-inflammatory cytokines such as TNFα and IL-1β [19]. Similar to p38, NF-κB is involved in the pathogenesis of AP and plays a vital role as an early and central event in the progression of inflammation [20]. And NF-κB activity was regulated by PAK1 [21].

PAK1 is a member of a serine/threonine protein kinase family, and plays a key role in multiple signal transduction pathways in mammalian cells. PAK1 is involved in multiple diseases, such as cancer, mental retardation, and allergy. PAK1 activation affects cell proliferation, cell apoptosis, and inflammation. The downstream of PAK1 is complex, and regulates multiple signal pathway, such as Aurora A [22], ILK [23], and MEK/ERK [24]. The function of PAK1 in cancer is well known, but its role in AP is still unclear.

In the present study, we investigated the PAK1 effects in AP mice and found that PAK1 up-regulated in cerulein-induced AP. To elucidate the mechanism of PAK1 in AP, NF-kB, and p38 signaling pathway were determined, and found that overexpression of PAK1 activated NF-kB and p38. Inhibition of PAK1 by shRNA or small molecule inhibitor FRAX597 decreased NF-kB and p38 activity, and alleviated the pathological damage in the pancreas, including decreasing the amylase and lipase levels in serum, and decreasing the levels of tumor necrosis factor-α, interleukin-6, and interleukin-1β. These results suggested that inhibition of PAK1 protects against AP by exerting anti-inflammatory by inhibiting NF-κB and p38 pathways. PAK1 is a potential therapy to alleviate AP patients in clinic.

## Materials and methods

### Animals

C57BL mice used in the present study are purchased from Slac (Shanghai, China). The mice were housed under laboratory conditions. The animals’ welfare and the experimental procedures were approved by the Animal Ethics Committee of Changzhou City No.1 People’s Hospital.

### AP model and experimental design

Cerulein (50 μg/kg) or saline (control) was given intraperitoneally every hour for six consecutive hours to the mice to induce AP [25]. FRAX597 (50 or 100 mg/kg) was administered by oral lavage at 1 h before the first injection of cerulein or saline. The mice were randomly allocated to four groups (*n*=5 for each group): (i) saline; (ii) cerulein (AP); (iii) saline+50 mg/kg FRAX597, and (iv) cerulein+100 mg/kg FRAX597. Eight hours after the final injection, the animals were killed, and the blood and pancreas were collected. All the blood samples were centrifuged and the serum was collected and stored at −80°C for measurement. The pancreas were rinsed with normal saline. One part of the pancreas was fixed in 10% formalin, others were frozen in liquid nitrogen and stored at −80°C.

### Adenoviral administration of AP model

A total of 15 mice were randomly divided into three groups: control group, adenoviral sh-NC treated AP mice group, and adenoviral sh-PAK1 treated AP mice group. After model establishment, mice were respectively treated by intraperitoneal injection with adenoviral sh-PAK1 (1.0 × 10^8^ PFU/ml/animal) and adenoviral sh-NC (1.0 × 10^8^ PFU/ml/animal). Seventy-two hours after the administration, the animals were killed, and collected the blood and pancreas. All of the blood samples were centrifuged and the serum was collected and stored at −80°C for measurement. The pancreas were rinsed with normal saline. One part of the pancreas was fixed in 10% formalin, others were frozen in liquid nitrogen and stored at −80°C.

### Lipase and amylase activity in serum

The serum were obtained from mice, and lipase and amylase activity assays were performed according to the manufacturer’s protocol of the amylase and lipase assay kits (Sigma, China).

### ELISA

Serum concentrations of TNF-α, IL-6, IL-1β, and IL-10 were determined via routine ELISA assay kits (R&D, Shanghai, China).

### Western blot

The cells were harvested by lysis buffer. The pancreatic tissues were lysed with RIPA lysis buffer containing 1 mmol/l PMSF. Then, the tissue homogenates were prepared and centrifuged at 12000 rpm for 10 min at 4°C. All protein sample concentration was determined by the BCA method. The proteins were separated by 10% of SDS/polyacrylamide gels, which were transferred to the PVDF membranes. The membranes were blocked in 5% milk for 1 h at 25°C, then incubated with primary antibodies overnight at 4°C. The next day, the membranes were washed with TBST three times, incubated with a horseradish enzyme-labeled secondary antibody. The ECL) reagents were added to visualize the chemiluminescence by ECL Plus detection system (Tanon, China). The band densities were analyzed with the ImageJ analysis system.

### Histological examination

The pancreas was fixed in 10% formalin, paraffin embedded, and Hematoxylin and Eosin stained. Multiple randomly were chosen from microscopic fields from five mice from each group. The mice were sentenced under isoflurane anesthesia. All the assessments were performed by an experienced pathologist blinded to the experimental design according to the criteria.

### Real-time PCR

Cells or pancreatic tissues were incubated with TRIzol reagent (Invitrogen Life Technologies, China) to achieve total RNA. For real-time PCR, 0.5 μg of total RNA was reverse-transcribed to synthesize cDNA using a first-strand cDNA synthesis kit. The quantitative real-time reverse transcriptase PCR was performed through the ABI PRISM 7500 Fast Sequence Detection System (Applied Biosystems, Shanghai, China) using the SYBR Green PCR kit. The mRNA primers for PAK1 is 5′-GTGTCTGAGACCCCAGCAGTA-3′ (forward primer) and 5′-GTGGTTCAATCACAGATCGTGT-3′ (reverse primer).

### Statistical analysis

Results were presented as mean ± S.E.M., and statistical analysis was performed using Prism GraphPad. All data were analyzed by one-way ANOVA. Differences were considered as significant at *P*-values <0.05.

## Results

### PAK1 up-regulated in cerulein-induced AP mice

**Figure 1:**
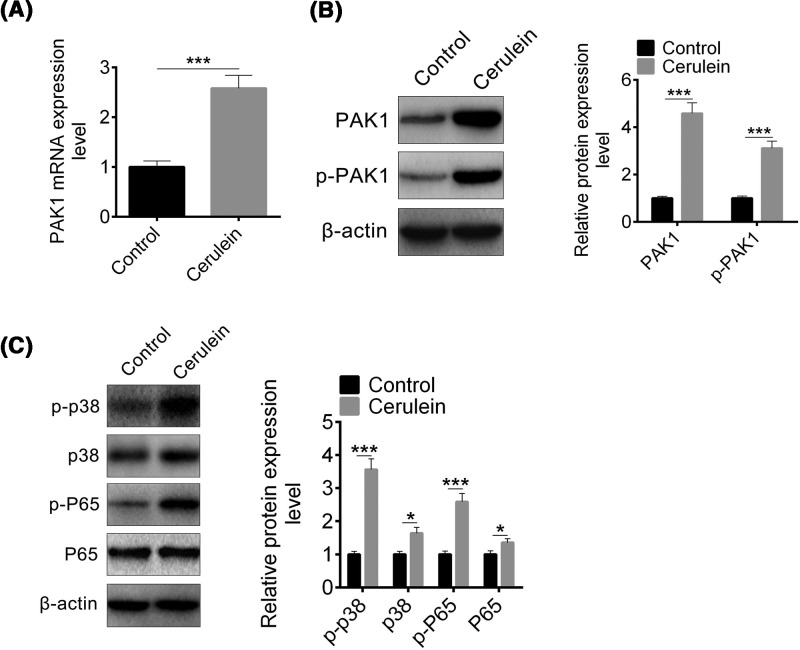
PAK1 up-regulated in cerulein-induced AP mice (**A**) mRNA expression levels of PAK1 in cerulein-induced AP mice detected by qRT-PCR. (**B**) The protein level of PAK1 and p-PAK1 in cerulein-induced AP mice detected by Western blot. (**C**) The protein level of p38, p-p38, p65, and p-p65 in cerulein-induced AP mice detected by Western blot. Data are presented as mean ± S.D.; *, *P*<0.05; ***, *P*<0.001, compared with control.

PAK1 is a serine/threonine kinase effector of the small Rho GTPases [26], and PAK1 activation stimulates NF-κB pathway [21]. We evaluated p-PAK1 protein and PAK1 expression in the pancreas of cerulein-induced AP by Western blot and qPCR, and found that *PAK1* mRNA level was increased in AP mice compared with control mice (Figure 1A). Consistent with mRNA level, both PAK1 protein and phosphorylation of PAK1 were up-regulated upon cerulein treatment (Figure 1B). As proinflammatory cytokines were increased in cerulein-induced AP mice, and led to MAPK signaling and NF-κB signaling activation, the phosphorylation of p38 and p65 were determined and found that both p38 and p65 phosphorylation were increased significantly (Figure 1C). These results indicated that PAK1 may be involved in AP.

### FRAX597 treatment in cerulein-induced AP mice alleviates pancreatitis symptoms

**Figure 2:**
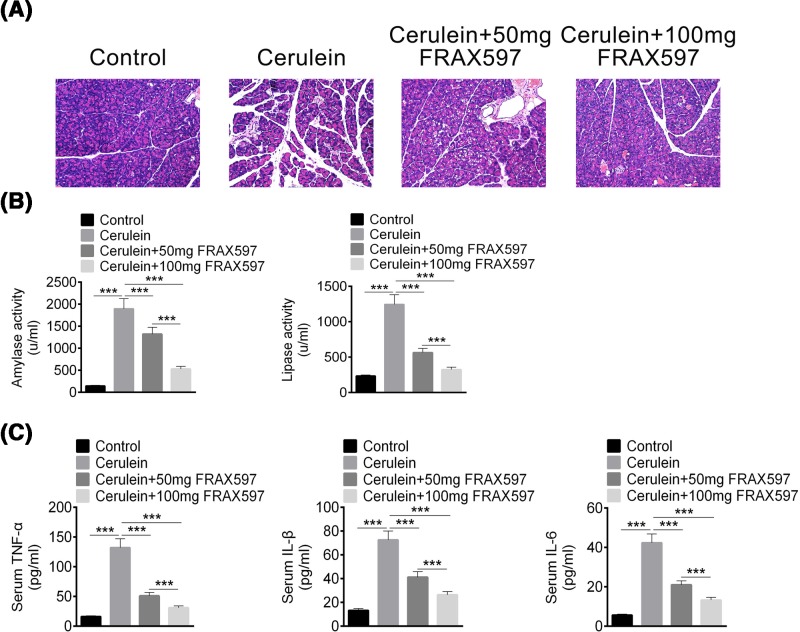
FRAX597 treatment in cerulein-induced AP mice alleviates pancreatitis symptoms (**A**) Representative pancreatic morphological changes in mice. (**B**) Serum amylase and lipase levels. (**C**) Serum TNF-α, IL-1β, and IL-6 levels. All data are presented as the mean ± S.D. (*n*=3). ***, *P*<0.001, compared with control.

As PAK1 was up-regulated in cerulein-induced AP mice, we hypothesized that PAK1 inhibition might have to function to alleviate pancreatitis symptoms. To prove this hypothesis, FRAX597, a potent, ATP-competitive inhibitor of PAKs [27], was used to treat AP mice at 50 and 100 mg/kg. Cerulein-induced AP mice displayed histological signs of AP characterized by necrosis of acinar cells and infiltration of inflammatory cells in pancreas (Figure 2A). Treatment with FRAX597 markedly alleviated the tissue damage (Figure 2A).

Serum amylase and lipase are two biomarkers to evaluate the development of AP [28]. Compared with the control group, cerulein increased the levels of amylase and lipase in serum significantly (Figure 2B). After treatment with FRAX597 at 50 and 100 mg/kg, the levels of lipase and amylase in serum were significantly decreased in cerulein-induced AP mice (Figure 2B). Except amylase and lipase, inflammatory mediators are important markers of AP model, and these inflammatory mediators exacerbate inflammation and tissue damage [29]. So we detected the inflammatory mediators, such as TNF-α, IL-1β, and IL-6 in AP mice to evaluate FRAX597 effect. The serum levels of TNF-α, IL-1β, and IL-6 were significantly elevated in cerulein-induced AP mice compared with the control group, and FRAX597 treatment significantly reduced these cytokines in both the 50 and 100 mg/kg FRAX597 groups (Figure 2C). These results indicated that PAK1 inhibition by FRAX597 alleviated pancreatitis symptoms by reducing amylase, lipase, and inflammatory mediators.

### PAK1 moduates p38 and NF-κB signaling pathway

As PAK1, p38, and NF-κB are activated in cerulein-induced AP mice, and PAK1 inhibition by FRAX597 alleviated pancreatitis symptoms, so we hypothesized that PAK1 played a role in AP through p38 and NF-κB pathway.

**Figure 3:**
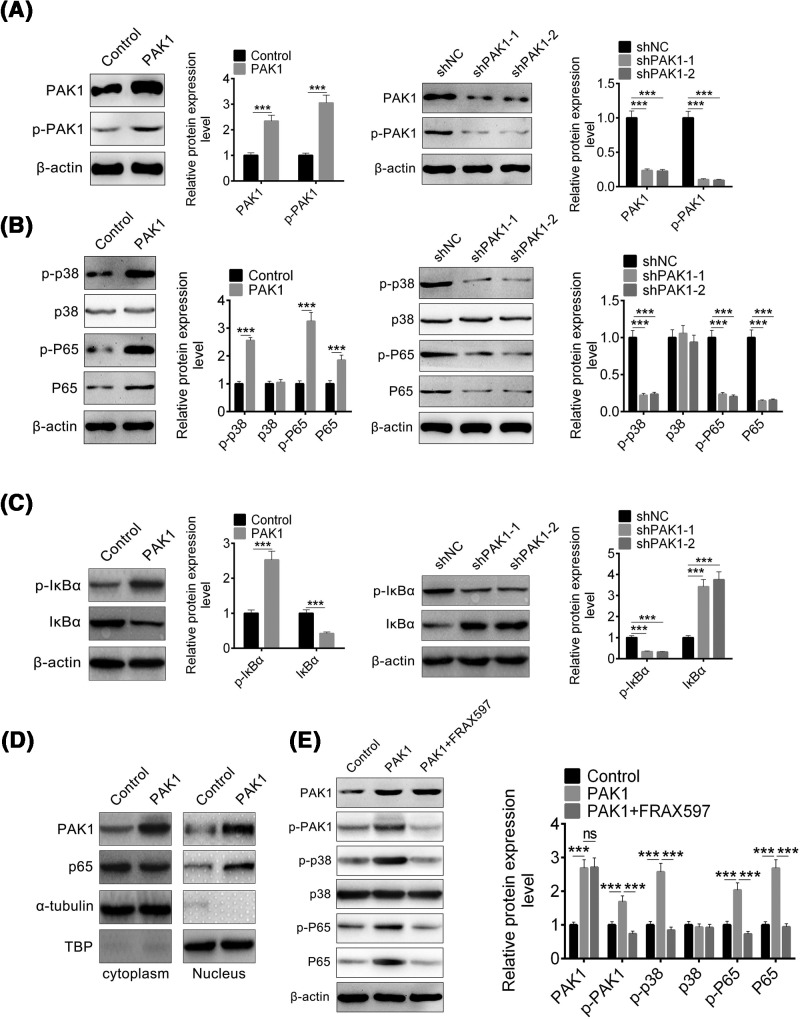
PAK1 moduates p38 and NF-kB signaling pathway (**A**) The protein level of PAK1 and p-PAK1 in primary pancreatic cells transfected with PAK1 (left) or sh-PAK1-1 and sh-PAK1-2 (right) for 48 h. (**B**) The protein level of p38, p-p38, p65, and p-p65 in primary pancreatic cells transfected with PAK1 (left) or sh-PAK1-1 and sh-PAK1-2 (right) for 48 h. (**C**) The protein level of IκBα and p-IκBα in primary pancreatic cells transfected with PAK1 (left) or sh-PAK1-1 and sh-PAK1-2 (right) for 48 h. (**D**) The protein level of PAK1 and p65 in primary pancreatic cells cytoplasmic and nuclear lysates transfected with PAK1. (**E**) The protein level of p38, p-p38, p65, and p-p65 in primary pancreatic cells transfected with PAK1 and treating with 1 μM FRAX597. All data are presented as the mean ± S.D. (*n*=3). NS, *P*>0.05; ***, *P*<0.001, compared with control. Each assay was performed in triplicate.

The p38 MAP kinases are a family of serine/threonine protein kinases, and p38α is involved in inflammatory responses, and regulates the biosynthesis of key proinflammatory mediators, such as TNF-a and IL-1b [5]. NF-κB is a nuclear transcription factor and regulates a series of transcription genes related to inflammation, and plays a critical role in AP [30]. The role of PAK1 on p38 and NF-κB pathway needs to be determined. First, PAK1 overexpression and knockdown system are constructed in primary pancreatic cells, and confirmed by Western blot. Upon PAK1 overexpression by plasmid transfection, both PAK1 and PAK1 phosphorylation protein were increased (Figure 3A). Meanwhile, PAK1 knockdown by shRNA led to a decrease in PAK1 and PAK1 phosphorylation protein level (Figure 3A). When PAK1 was overexpressed in primary pancreatic cells, p38 and p65 protein and its phosphorylation were determined, and found that p38 phosphorylation and p65 phosphorylation were up-regulated (Figure 3B; left). This indicated that PAK1 overexpression promoted p38 and NF-kB signaling pathway activation. We also found that p38 protein level had no obvious change while p65 protein level were increased significantly (Figure 3B; left). Then, p38 and p65 protein and its phosphorylation level were evaluated in PAK1 knockdown primary pancreatic cells, p38 phosphorylation and p65 phosphorylation were down-regulated, and p38 protein level had no obvious change while p65 protein level were decreased (Figure 3B; right). Binding of NF-κB to IκB proteins maintains NF-κB in an inactive state, and IκB phosphorylation leads to its degradation and NF-κB activation [21,31]. So the IκBα and IκBα phosphorylation level were detected, and found PAK1 overexpression promoted IκB phosphorylation while PAK1 knockdown inhibited IκB phosphorylation (Figure 3C). PAK1 overexpression also induced p65 nuclear translocation (Figure 3D). These results suggest that PAK1 activates p38 signaling by increasing p38 phosphorylation, while PAK1 activates NF-kB signaling by increasing p65 transcription, phosphorylation, and translocation.

To further confirm the PAK1 function on signaling pathway, PAK1 inhibitor FRAX597 was used to inhibit PAK1 activation. The cells were overexpressed by PAK1 plasmids and then treated with FRAX597, then PAK1, p38, and p65 protein levels were determined. The results showed that FRAX597 inhibited PAK1 phosphorylation, decreased p38 and p65 phosphorylation (Figure 3E). These results were similar to PAK1 knockdown, and proved that PAK1 led to p38 and NF-kB signaling pathway activation.

### FRAX597 reduces phosphorylation of PAK1, p38, and p65 *in vivo*

FRAX597 inhibited PAK1 phosphorylation and led to retard p38 and NF-kB signaling pathway *in vitro*. As FRAX597 treatment in cerulein-induced AP mice alleviated pancreatitis symptoms (Figure 2), so the effect of FRAX597 on phosphorylation of PAK1, p38, and p65 *in vivo* were evaluated.

**Figure 4:**
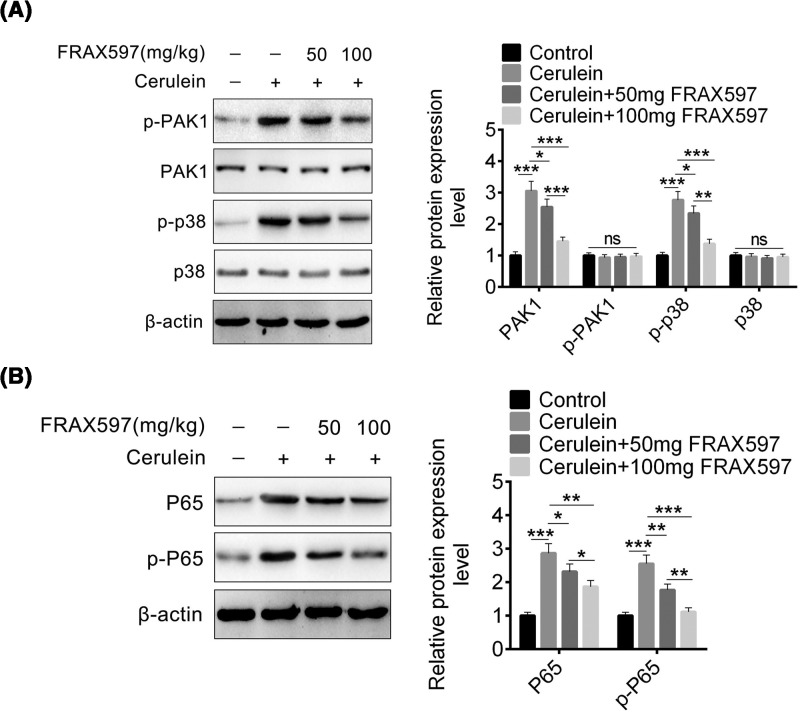
FRAX597 reduce phosphorylation of PAK1, p38 and p65 *in vivo* (**A**) The protein level of PAK1, p-PAK1, p38, and p-p38 in pancreas of cerulein-induced AP mice treated with 50 or 100 mg FRAX597 detected by Western blot. (**B**) The protein levels of p65 and p-p65 in pancreas of cerulein-induced AP mice treated with 50 or 100 mg FRAX597 detected by Western blot. All data are presented as the mean ± S.D. (*n*=3). NS, *P*>0.05;*,*P*<0.05; **, *P*<0.01; ***, *P*<0.001, compared with control. Each assay was performed in triplicate.

First, PAK1 protein and PAK1 phosphorylation level in the pancreas were determined, and found that phosphorylation of PAK1 was decreased upon 50 and 100 mg/kg FRAX597 in AP mice (Figure 4A). This suggested that FRAX597 treatment was successful. Then p38, p65 proteins and their phosphorylation levels in the pancreas were determined, and found that p38 protein was not changed while phosphorylation of p38 was decreased (Figure 4A). But both p65 expression and phosphorylation of p65 were decreased significantly (Figure 4B). These *in vivo* results were similar to the *in vitro* results, and indicated that FRAX597 treatment in cerulein-induced AP mice alleviated pancreatitis symptoms by down-regulating p38 and p65 signaling pathway.

### PAK1 knockdown by Ad-shPAK1 treatment in cerulein-induced AP mice alleviates pancreatitis symptoms

To further confirm PAK1 function in AP mice, PAK1 knockdown *in vivo* by adenovirus were constructed to evaluate PAK1 function in AP mice. Ad-shPAK1 and control adenovirus were administrated by intraperitoneal injection.

**Figure 5:**
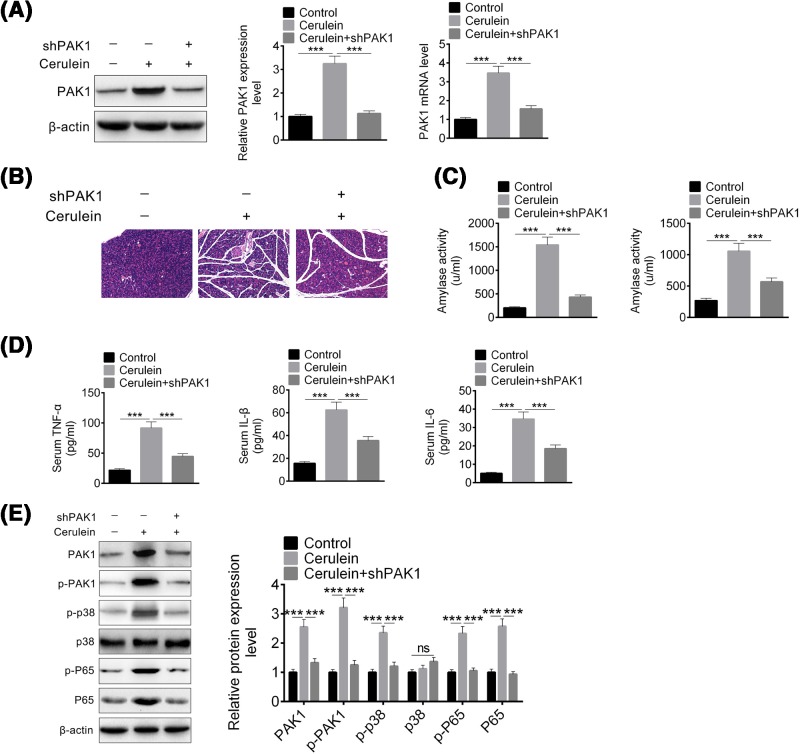
PAK1 knockdown by Ad-shPAK1 treatment in cerulein-induced AP mice alleviate pancreatitis symptoms (**A**) Protein and mRNA expression levels of PAK1 in pancreas of cerulein-induced AP mice detected by Western blot and qRT-PCR. (**B**) Representative pancreatic morphological changes in mice. (**C**) Serum amylase and lipase levels. (**D**) Serum TNF-α, IL-1β, and IL-6 levels. (**E**) The protein level of PAK1, p-PAK1, p38, p-p38, p65, and p-p65 in pancreas of cerulein-induced AP mice detected by Western blot and qRT-PCR. All data are presented as the mean ± S.D. (*n*=3). NS, *P*>0.05; ***, *P*<0.001, compared with control. Each assay was performed in triplicate.

First, the PAK1 knockdown efficacy was evaluated, and found that PAK1 protein level was decreased upon ad-shPAK1 injection. And the *PAK1* mRNA level was detected by qPCR, and in consistence with protein level (Figure 5A). As the model was constructed, the pancreatitis symptoms were evaluated. PAK1 knockdown alleviated histological signs of AP such as necrosis of acinar cells and infiltration of inflammatory cells in pancreatitis (Figure 5B). And the serum amylase and lipase were decreased after PAK1 knockdown *in vivo* (Figure 5C). Except amylase and lipase, inflammatory mediators in serum were also detected in pancreas of AP mice, and found that PAK1 knockdown inhibited TNF-α, IL-1β, and IL-6 release (Figure 5D). The phosphorylation of PAK1, p38, and p65 *in vivo* were also detected, and similar to FRAX597 treatment, PAK1 knockdown *in vivo* led to inhibition of PAK1, p38, and p65 phosphorylation (Figure 5E). These results showed that PAK1 knockdown *in vivo* alleviated pancreatitis symptoms by reducing amylase, lipase, and inflammatory mediators in cerulein-induced AP mice.

## Discussion

AP is a relatively common inflammatory disorder of the pancreas, it can lead to local and systemic complications. The pathophysiology of AP is always considered in three phases [32]. In the first phase, the trypsin is activated in pancreatic acinar cells, and leads to activate a variety of injurious pancreatic digestive enzymes and disrupt calcium signaling [33,34]. In the second phase, intrapancreatic inflammation is activated through multiple pathways, such as p38 and NF-κB [35]. In the last phase, extrapancreatic inflammation is activated, and leads to organ damage.

Many researchers have attempted to identify the initiation and aggravation of AP, but the disease is still poorly understood, and urgent to breakthrough to support effective treatment. Despite important studies have attempted to identify the pathogenesis of AP, the underlying mechanism has still poorly understood, and lacks sufficient clinic therapy to cure AP [36]. In this study, the function of PAK1 in AP is elucidated to help understand the initiation and aggravation of AP.

P21-activated kinases are a family of serine/threonine kinases and consist of two subgroups, which is Group I and Group II [37]. Group-I-PAKs (PAK 1–3) are well known, and play role in cell proliferation, cell apoptosis and inflammation. In Group-I-PAKs, PAK2 is ubiquitously expressed in different tissues, while PAK1 has a more reserved distribution [38]. In pancreas, PAK1 and PAK3 are expressed in pancreatic islets [39]. And PAK2 occurs in pancreatic acinar cells [38]. The role of Group-I-PAKs in AP is not well understood, the recent study showed that PAK2 is a mediator upon FAKs, MAPKs, and PI3K pathways, and PAK2 mediates trypsin activation and ROS production in pancreatic acini, and leads to pancreatic acini apoptosis and necrosis [40]. But the role of PAK1 in AP remains unknown. NF-κB and p38 MAPK are the relevant kinases involved in inflammatory responses, and regulate the biosynthesis of key proinflammatory mediators [19]. In the progress of AP initiation and aggravation, inflammation including intrapancreatic and extrapancreatic inflammation is activated to cause organ dyfunction. It is obvious that NF-κB and p38 are involved in AP [6,7]. Some studies showed that PAK1 regulated p38 activity [8,9] and NF-κB activity (2121).

Therefore, we hypothesize that PAK1 has function in AP development, and the results showed that PAK1 and p-PAK1 protein level were up-regulated in cerulein-induced AP. The expression level of PAK1, p38, and p65 was analyzed by Western blot. The results showed that PAK1 inhibition by shRNA or small molecule inhibitor FRAX597 decreased NF-kB and p38 activity, thus we speculated that the decreased NF-kB and p38 activity mediated PAK1 function to alleviated the pathological damage in the pancreas, including decreasing the amylase and lipase levels in serum, decreasing the levels of tumor necrosis factor-α, interleukin-6, and interleukin-1β. These results suggested that PAK1 inhibition protects against AP by exerting anti-inflammatory effect by inhibiting NF-κB and p38 pathways. PAK1 inhibition is a potential therapy to alleviate AP and exhibits great potential as a therapeutic target to in clinic for AP patients.

## Abbreviations

AP: acute pancreatitis
FAK: Focal Adhesion Kinase
IκB: I-Kappa B
IL: Interleukin
MAP: Mitogen-Acivated Protein
MAPK: Mitogen-Activated Protein Kinase
NF-κB: Nuclear Factor Kappa B
PAK1: P21-activated kinase 1
PFU: Plaque Forming Unit
PI3K: Phosphatidylinositol 3 Kinase
qPCR: Quantitative Polymerase Chain Reaction
RIPA: Radioimmunoprecipitation assay
ROS: Reaction Oxygen Species
TBST: Tris Bufferred saline Tween
TNF: Tumor Necrosis Factor
